# Speech-based early depressive symptom screening using MAX-Net: a multi-scale autoencoder-augmented xLSTM framework

**DOI:** 10.3389/fpsyt.2026.1907554

**Published:** 2026-09-03

**Authors:** Zhiguo Zheng, Lijuan Liang, Jin Zhang, Guanjun Wang, Chenyang Xue

**Affiliations:** 1 School of Information and Communication Engineering, Hainan University, Haikou, China; 2 School of Electronic Science and Technology, Hainan University, Haikou, China; 3 Department of Psychology, The First Affiliated Hospital of Hainan Medical University, Haikou, China; 4 School of Information Engineering, Hainan Vocational University of Science and Technology, Haikou, China; 5 School of Intelligent Medicine and Technology (Big Data Research Center), Hainan Medical University, Haikou, China

**Keywords:** data augmentation, deep learning, depressive symptoms, small-sample learning, speech analysis

## Abstract

**Background:**

Early screening for depressive symptoms lacks objective and stable biomarkers, while traditional methods based on questionnaires and subjective assessments suffer from issues such as low efficiency. Although the use of artificial intelligence to analyze speech signals has become a growing trend, existing methods are limited by challenges such as small medical datasets, complex features, and long-term temporal dependencies, resulting in insufficient recognition accuracy and poor cross-dataset generalization performance.

**Methods:**

To address the above issues, this paper proposes MAX-Net, a multi-scale feature-enhanced long-term sequential autoencoder network based on an improved extended long short-term memory (xLSTM) for depressive symptom screening. MAX-Net combines the improved xLSTM with autoencoder feature enhancement and introduces a Multi-Scale Convolutional layer (MSC) module. To mitigate the issue of data sparsity, we propose an autoencoder-based feature space enhancement strategy. Furthermore, we investigate the impact of varying numbers of augmented samples on model performance. The model is trained and evaluated on the in-house dataset HMU-DDAC-24 and retrained on the public dataset MODMA using the same architecture and hyperparameters to assess its stability and cross-dataset applicability.

**Results:**

Experiments on HMU-DDAC-24 show that MAX-Net achieves an Area Under the ROC Curve (AUC) of 0.945 and a Mean Absolute Error (MAE) of 1.61 in the depression symptom classification task, significantly outperforming existing baseline models. It maintains competitive performance on the MODMA dataset (AUC = 0.804), suggesting architectural stability and consistent effectiveness across different data sources when retrained. Further experiments revealed that the optimal data augmentation strategy varies across different datasets: on HMU-DDAC-24, performance peaks when 15,000 samples are generated, whereas on MODMA, performance stabilizes when 18,000–22,000 samples are generated.

**Conclusion:**

MAX-Net provides an effective framework for speech-based early depression screening and demonstrates consistent performance across datasets under a unified configuration. These findings indicate that the proposed architecture is robust to variations in data distribution. Furthermore, the autoencoder-based augmentation strategy has proven beneficial in low-data-volume scenarios. Overall, this study supports the application potential of MAX-Net as an auxiliary tool for early depression screening.

## 1 Introduction

### 1.1 Background

Depression has become a common mental disorder affecting individuals’ physical and mental health, impacting hundreds of millions of people worldwide and placing a heavy burden on both individuals and society (1, 2). In clinical practice, screening still relies primarily on subjective rating scales such as the HAMD and PHQ-9 (3). While these tools are widely used, they are time-consuming and susceptible to rater bias. Furthermore, there is currently a lack of reliable, objective biomarkers, a limitation that makes large-scale, efficient early screening difficult to conduct.

### 1.2 Progress of other research methods

To address these issues, researchers have explored various methods, including electroencephalography (EEG), facial expression analysis, and functional near-infrared spectroscopy (fNIRS) (4–9). While these methods can provide useful physiological information, in many cases they require specialized equipment and controlled environments, which increases costs and operational complexity, thereby limiting their potential for application in large-scale routine screening.

### 1.3 Current status and potential of speech analysis in screening for depressive symptoms

Compared to the aforementioned methods, voice data collection is more convenient and does not require invasive procedures, making voice-based analysis more suitable for real-world and large-scale scenarios. Previous studies have reported that depressive symptoms is associated with changes in acoustic features such as prosody, pitch variation, and speech rate (10–12). In recent years, with breakthroughs in deep learning for speech and emotion recognition, research on speech-based depressive symptom screening using models such as CNNs, LSTMs, and Transformers has made initial progress (13, 14).

Despite these advances, several practical challenges remain. Due to privacy constraints and difficulties in data collection, clinical speech datasets are typically small and class-imbalanced, leading to a high risk of model overfitting. Furthermore, depressive speech patterns exist across multiple time scales, ranging from short-term spectral changes to longer-duration prosodic structures. Capturing these patterns simultaneously within a single framework is challenging. Another issue lies in modeling long-range dependencies, as relevant cues may appear sparsely in long speech segments, and their influence diminishes as the sequence length increases.

To address these issues, we propose MAX-Net, a multi-scale feature enhancement network for speech-based depressive symptom screening. The model combines multi-scale convolutional modules for local feature extraction with an extended LSTM (xLSTM) for modeling long-term dependencies. We further introduce an autoencoder-based (15) feature enhancement strategy to mitigate data sparsity. Additionally, we analyze the relationship between the scale of enhancement and model performance from a bias-variance perspective.

### 1.4 Research objectives and novel contributions of this work

To address the core challenges of limited sample size, weak long-sequence modeling, and complex features in depressive symptom recognition from speech signals, we propose MAX-Net, a multi-scale feature enhanced long-sequence autoencoder network for depressive symptom detection, aiming to significantly improve recognition accuracy and model generalization.

The contributions and innovations of this study are fourfold:

1. Modeling innovations for small samples and complex features: We propose a Multi-Scale Convolutional Layer (MSC) module that employs parallel convolutions to capture local patterns of speech signals at varying temporal scales. In parallel, we design an autoencoder (AE) based on a modified U-Net architecture, which enhances feature robustness and augments training samples through a feature reconstruction enhancement strategy, effectively alleviating data scarcity.
2. Architectural innovations and module synergy for long-sequence dependencies: Unlike existing works that rely solely on a single-branch sLSTM in the local fusion layer, we adopt Xlstm(comprising both sLSTM and mLSTM branches) as the core backbone, achieving complementary advantages with the MSC module. The high-dimensional local distortion features extracted by MSC are directly fed as inputs to xLSTM, addressing the lack of local microscopic receptive fields in conventional temporal models. Meanwhile, the matrix memory of mLSTM mitigates information decay over long speech sequences.
3. Systematic data augmentation strategy and theoretical analysis: We propose a comprehensive data augmentation and training mechanism tailored for small-sample medical scenarios. Through both experimental and theoretical analyses (bias-variance trade-off), we reveal that model performance reaches an optimum when the number of AE-generated samples is at a specific proportion relative to the original training samples, thereby providing quantifiable guidelines for data augmentation in similar medical speech settings.
4. High-performance framework for early screening and empirical evaluation: We integrate all the above components into a unified MAX-Net framework and conduct systematic evaluations on HMU-DDAC-24, a real-world speech dataset collected from college students covering both healthy individuals and those with depressive symptoms (PHQ-9 ≥ 5). Our model achieves superior performance in binary depressive symptom classification, significantly outperforming multiple baseline models, thus demonstrating its substantial potential for early screening in subclinical high-risk populations.

The remainder of this paper is organized as follows. Section 2 reviews related work. Section 3 describes the proposed method. Section 4 presents the experimental results. Section 5 discusses the findings. Section 6 concludes the paper.

## 2 Related work

### 2.1 Speech-based approaches for depressive symptom screening

Speech is an easily accessible, non-invasive biomarker of depressive symptoms. Previous studies have consistently reported differences in acoustic features, such as speech rate, fundamental frequency, intensity, and prosodic variability, between individuals with depressive symptoms and healthy controls (16–18). Early methods relied on manually designed features (MFCCs) and traditional classifiers (such as support vector machines (SVMs) or logistic regression (19)); these approaches were heavily dependent on expert prior knowledge and struggled to capture complex, nonlinear speech patterns. Deep learning enables end-to-end learning of more discriminative representations from raw or shallow features. Several mainstream architectures have emerged, including CNNs, LSTMs, hybrid models combining both [e.g., ConvBiLSTM (20), DepAudioNet (21)], and Transformer-based models [e.g., HATN (22), MLA (23)], which are used for local, temporal, and long-range context modeling, respectively.

### 2.2 Data augmentation techniques

The scarcity of high-quality medical data is the primary bottleneck limiting the development of models for depressive symptoms screening. Therefore, expanding the dataset using data augmentation techniques is crucial for improving the models’ generalization ability (24–26). Data augmentation methods can be broadly categorized into two types: signal transformation methods and deep generative models. The former include techniques such as adding noise and temporal and frequency masking in SpecAugment (27, 28); the latter involve Generative Adversarial Networks (GANs) for speech/physiological signal synthesis (29, 30), as well as diffusion models capable of generating high-fidelity, emotionally controllable audio (31, 32). In speech-based depressive symptom analysis, effective augmentation must address the challenges of small sample sizes and high inter-individual variability (33), making the generation of controllable pathological speech a key research direction in resource-constrained clinical settings (34).

### 2.3 Limitations and research motivation

Despite the progress made, several key challenges continue to hinder the practical clinical application of speech-based depressive symptom screening.

1. Existing sequence models (such as LSTM- or CNN–LSTM-based architectures) still suffer from information decay and insufficient long-range context modeling capabilities. They often struggle to capture sparsely distributed and subtly expressed depressive symptom-related cues in long speech segments, which is particularly critical for screening early-stage or mild depressive symptoms.
2. Many studies rely on fixed-scale feature representations (such as MFCCs or Mel-frequency spectrograms), failing to fully exploit the dynamic patterns of speech across multiple time windows and frequency bands. This lack of multis-cale modeling limits the ability to characterize variations in speech rate, prosody, and intensity trajectories, thereby weakening the discriminative power of the features.
3. Clinical datasets are small and class-imbalanced, making deep models prone to overfitting. This leads to unstable model performance and difficulty in generalizing to new recording environments, institutions, or populations.
4. Many methods are evaluated primarily on staged or interview-based corpora (e.g., DAIC-WOZ (35)), which differ from spontaneous speech in real clinical settings; their effectiveness in actual early screening scenarios remains inadequately validated.

These limitations collectively highlight the urgent need for a unified framework that can simultaneously address multiscale feature extraction, long sequence modeling, and data scarcity in clinically realistic early screening environments, thereby providing a scalable pathway for speech based early screening technologies.

## 3 Materials and methods

### 3.1 Participants

A total of 143 undergraduate students (aged 18–22) from Hainan Medical University were recruited for this study. Participants were grouped based on their Patient Health Questionnaire-9 (PHQ-9) scores (36): a healthy control group (PHQ-9< 5) and a depressive symptom group (PHQ-9 scores ≥ 5), with a focus on individuals in the early stages of depressive symptom who had not yet received pharmacological treatment. All participants signed informed consent forms, and this study was approved by the Ethics Committee of the First Affiliated Hospital of Hainan Medical University (Ethics Approval No.: 2024-KYL-133) and was conducted in accordance with the Declaration of Helsinki.

We constructed the HMU-DDAC-24 speech corpus, comprising four tasks to elicit varied emotional states: (1) Neutral Reading of a standard passage; (2) Daily-life Q&A; (3) Positive Recall; and (4) Negative Recall. Recordings were made in an acoustically controlled environment.

### 3.2 Data preprocessing and feature extraction

**Figure 1:**
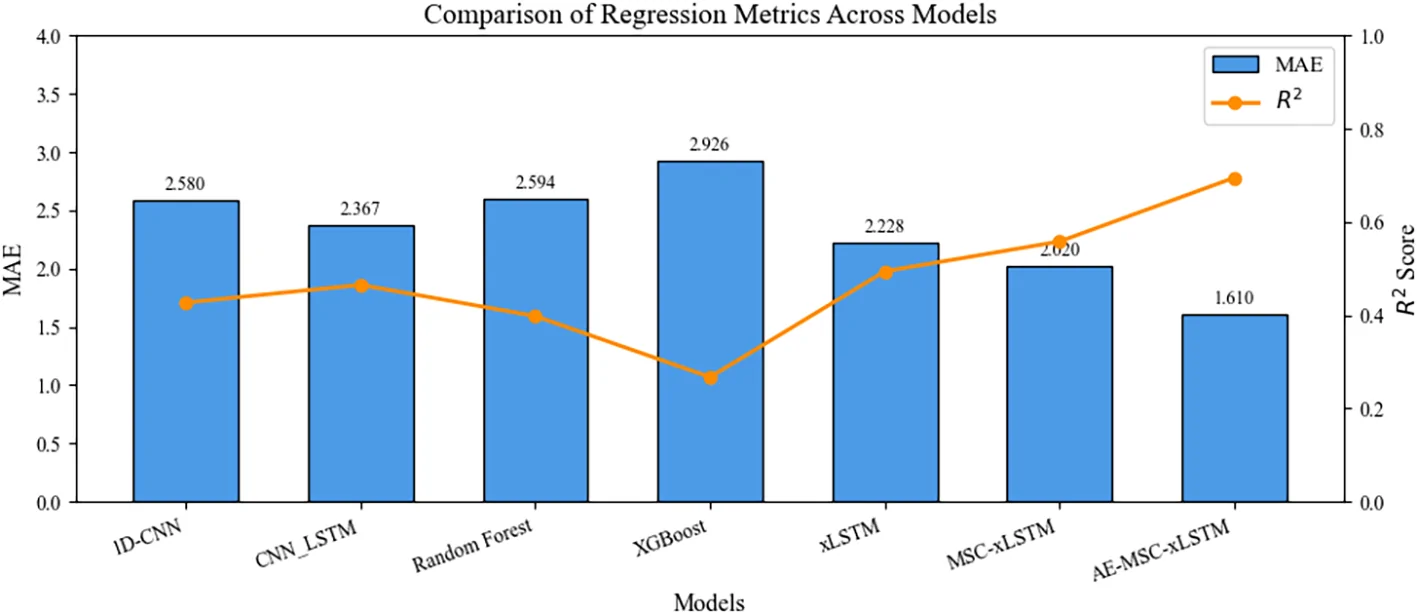
Comparison of speech signals before and after Wiener filtering.

**Figure 2:**
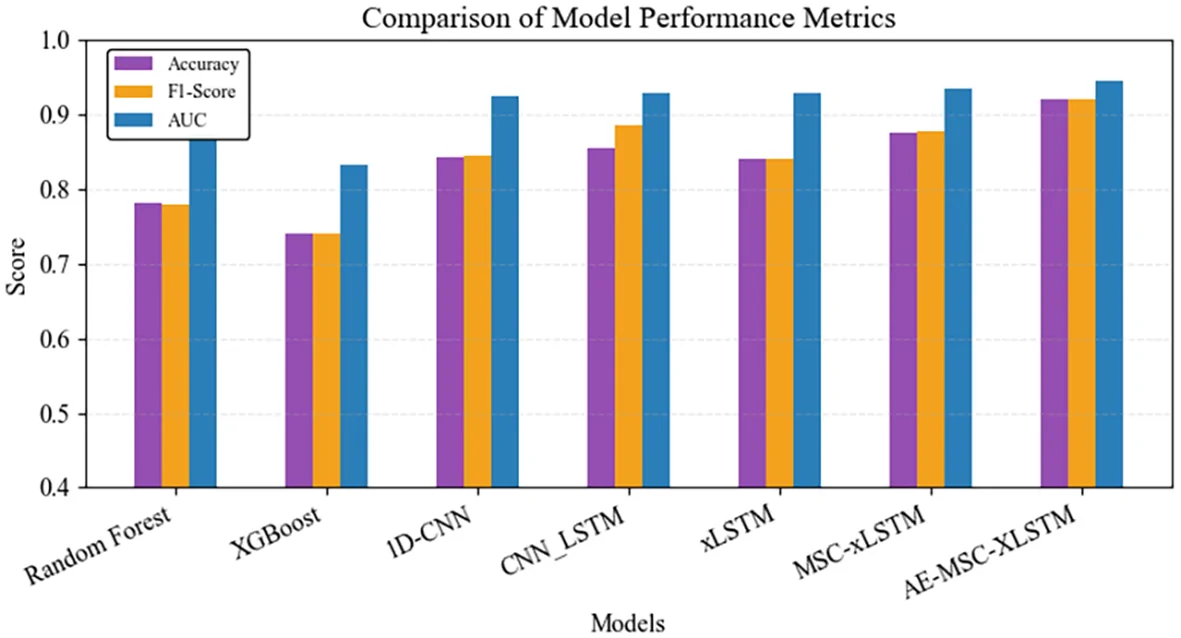
Speech signal before and after silence removal using voice activity detection (VAD).

Raw audio was preprocessed through denoising via Wiener filtering (37), silence removal, resampling to 16 kHz, and segmentation into 1s frames, yielding 10,165 frames for analysis. (See **Figures 1**, **2** for examples).

A 473-dimensional feature set was extracted, containing 445 frame-level and 28 utterance-level statistical features (**Table 1**). Frame-level features included a 128-dim log-Mel spectrogram, a 257-dim linear spectrogram, and 60-dim enhanced MFCCs (with delta and delta-delta coefficients) (38–40). Features comprised statistics (e.g., mean, std.) of fundamental frequency (F0), zero-crossing rate, and short-time energy. All features were Z-score normalized.

**Table 1:** Summary of audio feature dimensionality.

Feature type	Subtype	Dimension	Description
Frame-Level Features (445 dimensions)	Mel-Spectrogram Features	128	Log-Mel spectrogram extracted using 128 Mel filterbanks (n_mels_ = 128)
	Linear Spectrogram Features	257	Linear spectrogram magnitudes based on short-time Fourier transform (n_fft_ = 512, 257 frequency bins)
	Enhanced MFCC Features	60	Base MFCC: 20 + Δ: 20 + ΔΔ: 20
Statistical Features (28 dimensions)	Fundamental Frequency F0 Statistics	8	Mean, standard deviation, median, 25th and 75th percentiles, voiced segment ratio, skewness, kurtosis
	Zero-Crossing Rate (ZCR) Statistics	6	Mean, standard deviation, median, 25th and 75th percentiles, skewness
	Short-Time Energy Statistics	6	Mean, standard deviation, median, 25th and 75th percentiles, skewness
Total	-	473	445 frame-level + 28 statistical features

The dataset was split 8:2 at the participant level (training:test), preserving a ~50:50 label ratio to prevent data leakage. All reported performance metrics were computed at the participant level, where predictions from the four recording sessions for each participant were aggregated to yield a single outcome per participant.

### 3.3 MAX-Net architecture

**Figure 3:**
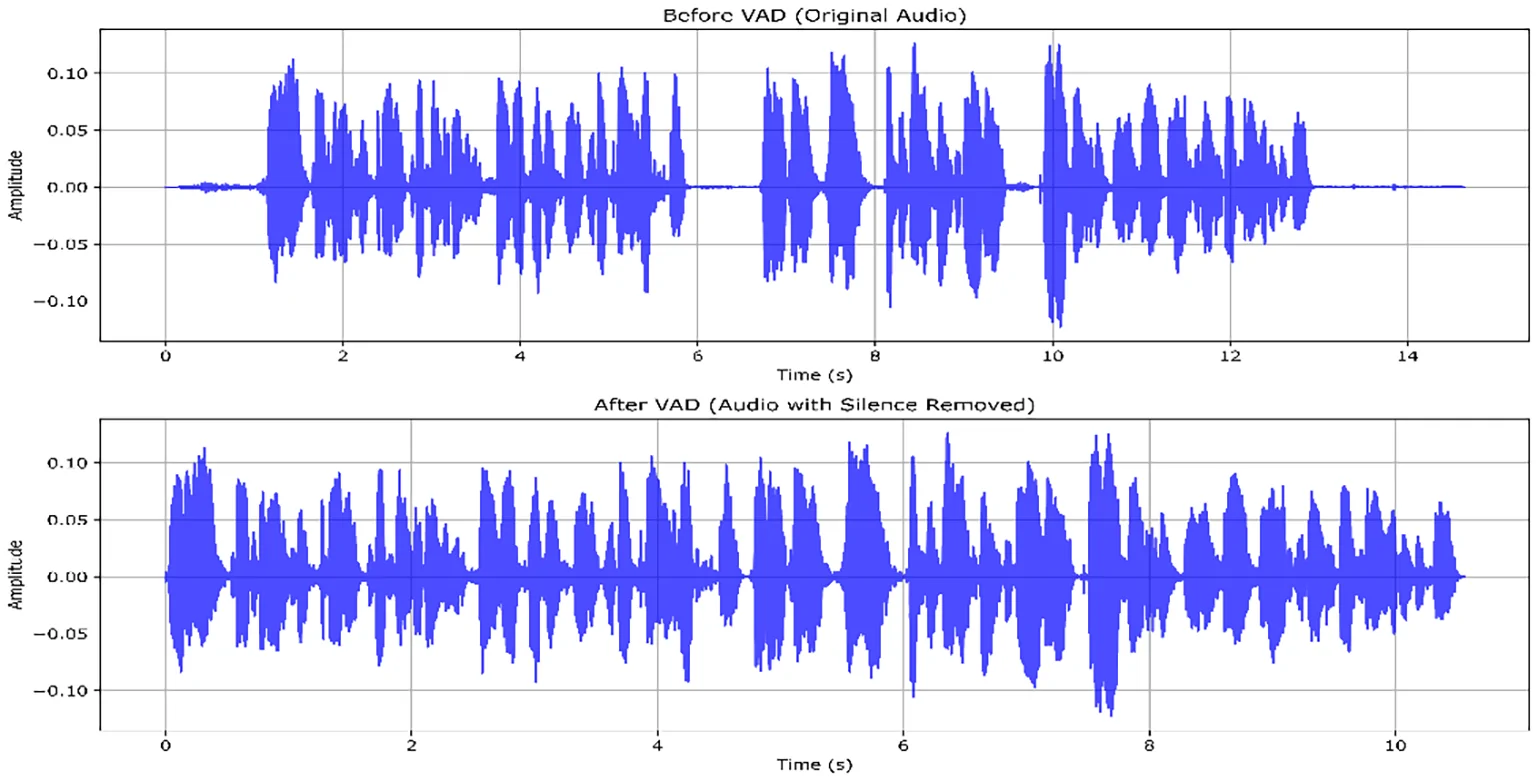
Overall framework of the speech data processing pipeline.

The proposed MAX-Net integrates three core components (**Figure 3**): a feature enhancement AE, a MSC (41) module, and a xLSTM network for temporal modeling.

#### 3.3.1 Feature enhancement module

**Figure 4:**
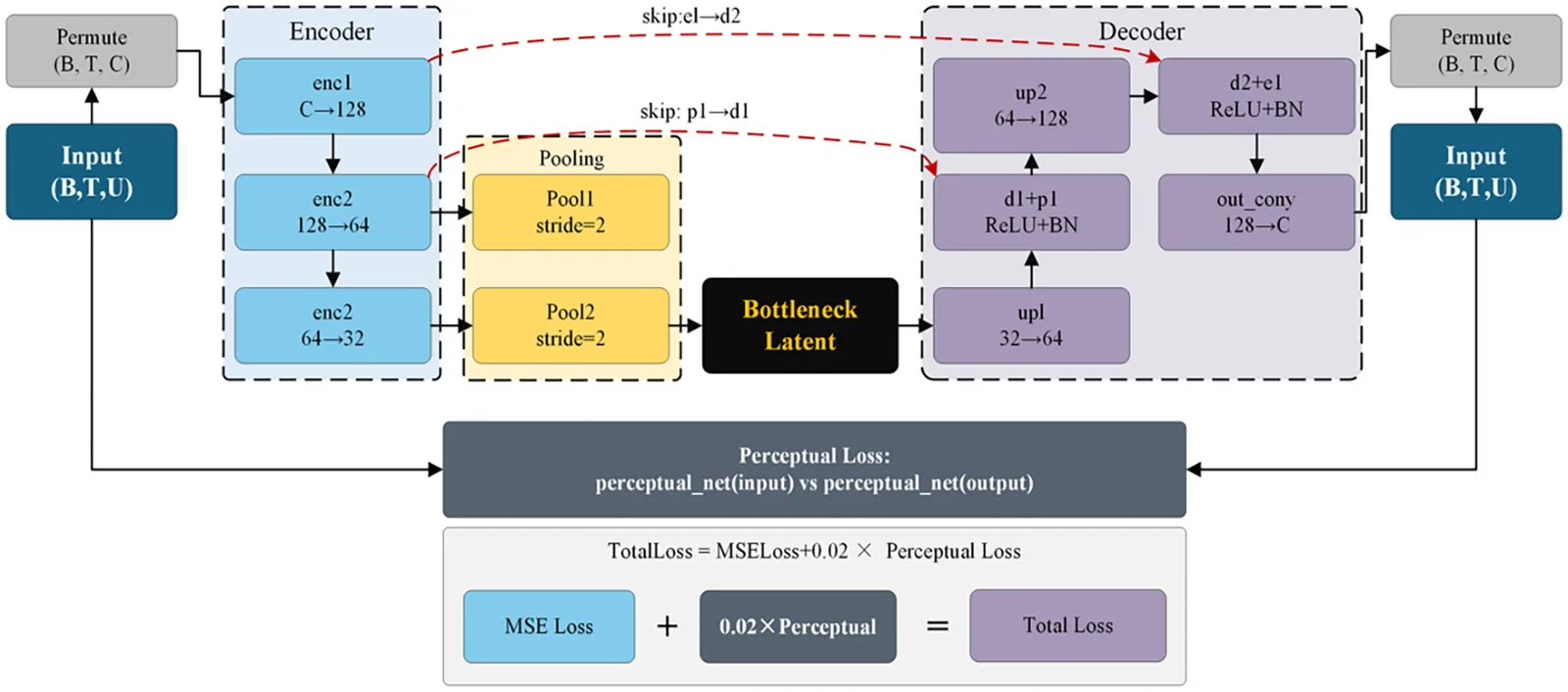
Architecture and parameter configuration of the proposed improved autoencoder.

An improved convolutional AE with U-Net-like skip connections (42) reconstructs input features. Its loss combines reconstruction and perceptual losses:


$${ℒ}_{AE}=\underset{\text{Reconstruction Loss }}{\underset{︸}{{\left\|\hat{X}-X\right\|}_{2}^{2}}}+\lambda_{p}\cdot \underset{\text{Perceptual Loss }}{\underset{︸}{{\left\|\phi \left(\hat{X}\right)-\phi \left(X\right)\right\|}_{2}^{2}}},$$

where *λ_p_* = 0.02 and *ϕ(·)* is a perceptual subnetwork (**Figure 4**).

#### 3.3.2 Backbone network: AE–MSC–xLSTM architecture

**Figure 5:**
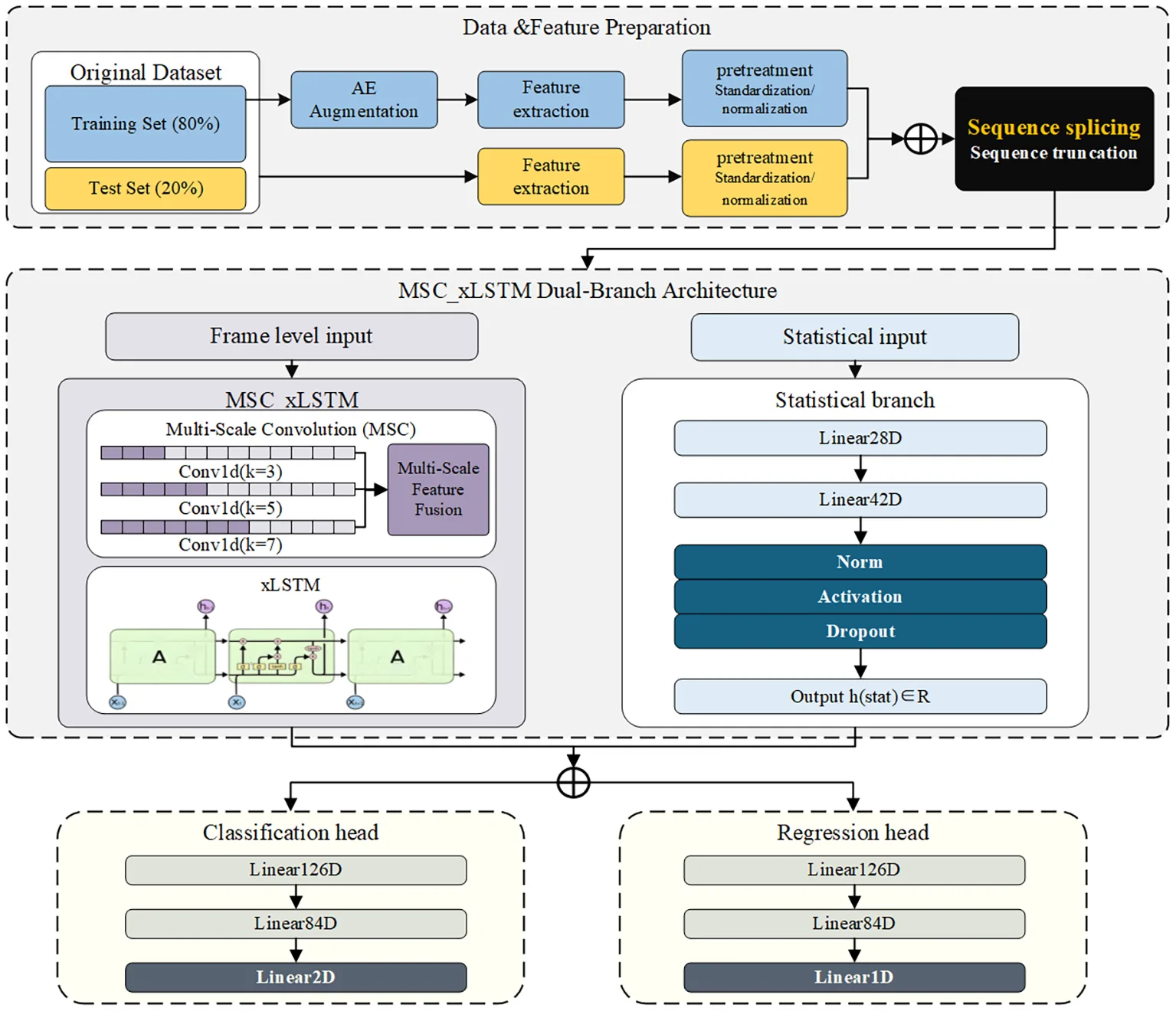
Architecture and parameter configuration of the proposed AE–MSC–xLSTM (MAX-Net) model.

Enhanced features pass through an MSC module and an xLSTM (**Figure 5**).

1. MSC module: Employs parallel 1D convolutions with kernel sizes {3, 5, 7} to capture multi-scale local patterns. Outputs are concatenated:

$$H_{\text{MSC}}=\left[H^{\left(3\right)};\;H^{\left(5\right)};\;H^{\left(7\right)}\right]\in {\mathbb{R}}^{T\times d}$$

2. xLSTM-based temporal modeling: An xLSTM module (43) (one sLSTM + one mLSTM block) with exponential gating and matrix memory processes H_LSTM_ to model long-term dependencies:

$$H_{\text{LSTM}}=\text{xLSTM}\left(H_{\text{MSC}}\right)$$

3. Output layer and multi-task learning: Temporal features are aggregated via global average pooling. Separate MLP heads perform binary depressive symptoms classification (cross-entropy loss) and severity regression (mean squared error).

### 3.4 Model parameters and training configuration

The AE was pre-trained for 1000 epochs and the main MAX-Net for 100 epochs, both with Adam optimization; other hyperparameters are listed in **Table 2**.

**Table 2:** Model architecture and training hyperparameters.

Component	Parameter	Value & description
Input Settings	Input Feature Type	Acoustic features (473 dimensions)
	Audio Sampling Rate	16 kHz
AE Module	Architecture	Improved convolutional autoencoder (U-Net–like with skip connections)
	Encoder Channels	128 → 64 → 32
	Decoder Channels	32 → 64 → 128
	Perceptual Loss Weight (*λ_p_*)	0.02
MSC Module	Convolution Kernel Sizes	[3, 5, 7]
	Channels per Kernel	32 per kernel size (total output dimension = 96)
xLSTM Module	Structure	One sLSTM block + one mLSTM block
	Hidden Size	32
Output Layer	Classification Output Dimension	2 classes (healthy vs. depressive symptoms)
	Regression Output Dimension	1 (normalized depressive symptoms severity score)
Training Settings	Batch Size	512
	Optimizer	Adam
	AE Learning Rate	1 × 10^−3^
	Main Model Learning Rate	5 × 10^−4^
	Number of Epochs	1000 (AE), 100 (main model)
	Classification Loss Function	Cross-entropy loss
	Regression Loss Function	Mean squared error (MSELoss)
	Gradient Clipping	max_norm = 1.0
	AE Learning Rate Scheduler	StepLR (decay by 0.5 every 200 epochs)

The AE module is used for speech feature enhancement. Its reconstructed outputs are treated as augmented training data and incorporated into the training of the MSC–xLSTM backbone, thereby improving the robustness and generalizability of the overall MAX-Net model under data-scarce conditions.

### 3.5 Validation on the MODMA dataset

To further validate the stability and cross-dataset applicability of the MAX-Net architecture, we retrain the model on the speech subset of the MODMA dataset (44) using the same architecture and hyperparameters as described earlier. This dataset was released by Lanzhou University in 2015, and we utilize the speech data from 52 participants. This does not imply zero-shot cross-dataset generalization, but rather shows that the architecture is robust to variations in data distribution when retrained. To further assess the actual cross-dataset generalization capability, we additionally conducted a zero-shot transfer experiment by directly applying the HMU-DDAC-24-trained model to the MODMA test set without retraining. The results (AUC = 0.586, see **Supplementary Table 1**) confirm the expected performance drop due to significant domain gaps between the two datasets (e.g., recording conditions, language, and task paradigms), further justifying the need for task-specific retraining in real-world deployment scenarios.

## 4 Experiment design and results

### 4.1 Experimental setup

The experiment was conducted using the HMU-DDAC-24 dataset (143 participants, 572 samples). Participants were divided into a depressive symptom group (PHQ-9 ≥ 5, n = 71) and a healthy control group (PHQ-9< 5, n = 72) based on their PHQ-9 scores for the binary classification task; continuous PHQ-9 scores were used for severity regression. The demographic characteristics and score distributions of the dataset are summarized in **Table 3**.

**Table 3:** Participants and score distribution.

Group	PHQ-9 score range	Number	Male	Female
HC	0–4	72	32	40
Mild Symptoms	5-9	42	19	23
Moderate Symptoms	10-14	29	15	14

To assess generalization ability, we employed a strict 80:20 training-test split by participant, combined with stratified sampling to maintain a consistent depressive symptom-to-healthy ratio in both sets. During splitting, we ensured that all speech segments from the same participant were assigned to the same set to avoid data leakage caused by segments from the same individual being scattered across the training and test sets, thereby ensuring the reliability of the evaluation results.

Model performance was evaluated from two perspectives: predictive accuracy and practical efficiency. For classification tasks, we report accuracy, precision, recall, F1 score, and area under the ROC curve (AUC); for regression tasks, we report the coefficient of determination (R²), Mean Absolute Error (MAE), and Root Mean Squared Error (RMSE). Efficiency is measured by the number of trainable parameters, training time, and inference time per sample, while generalization ability is reflected by the performance gap between the validation and test sets.

### 4.2 Performance of MAX-Net against baselines

We evaluated and compared our proposed MAX-Net with traditional machine learning methods (Random Forest, XGBoost) and deep learning baseline models (1D CNN, CNN-LSTM, xLSTM, MSC-xLSTM). As shown in **Table 4**, MAX-Net achieved the best performance across all key metrics.

**Table 4:** Comparison of experimental results for each model.

Model	Accuracy	F1-score	AUC	MAE	R²
Random Forest	0.781 ± 0.009	0.780 ± 0.009	0.874 ± 0.006	2.594 ± 0.024	0.398 ± 0.008
XGBoost	0.741 ± 0.008	0.741 ± 0.009	0.859 ± 0.007	2.926 ± 0.037	0.267 ± 0.004
1D CNN	0.844 ± 0.005	0.846 ± 0.005	0.917 ± 0.001	2.580 ± 0.036	0.427 ± 0.016
CNN–LSTM	0.856 ± 0.003	0.886 ± 0.003	0.920 ± 0.003	2.367 ± 0.044	0.465 ± 0.012
xLSTM	0.842 ± 0.005	0.842 ± 0.005	0.928 ± 0.004	2.228 ± 0.060	0.494 ± 0.017
MSC–xLSTM	0.875 ± 0.005	0.878 ± 0.005	0.931 ± 0.005	2.020 ± 0.085	0.558 ± 0.029
MAX-Net(AE–MSC–xLSTM)	0.901 ± 0.004	0.902 ± 0.004	0.945 ± 0.003	1.610 ± 0.022	0.695 ± 0.027

All results are reported as mean ± standard deviation over repeated runs.

**Figure 6:**
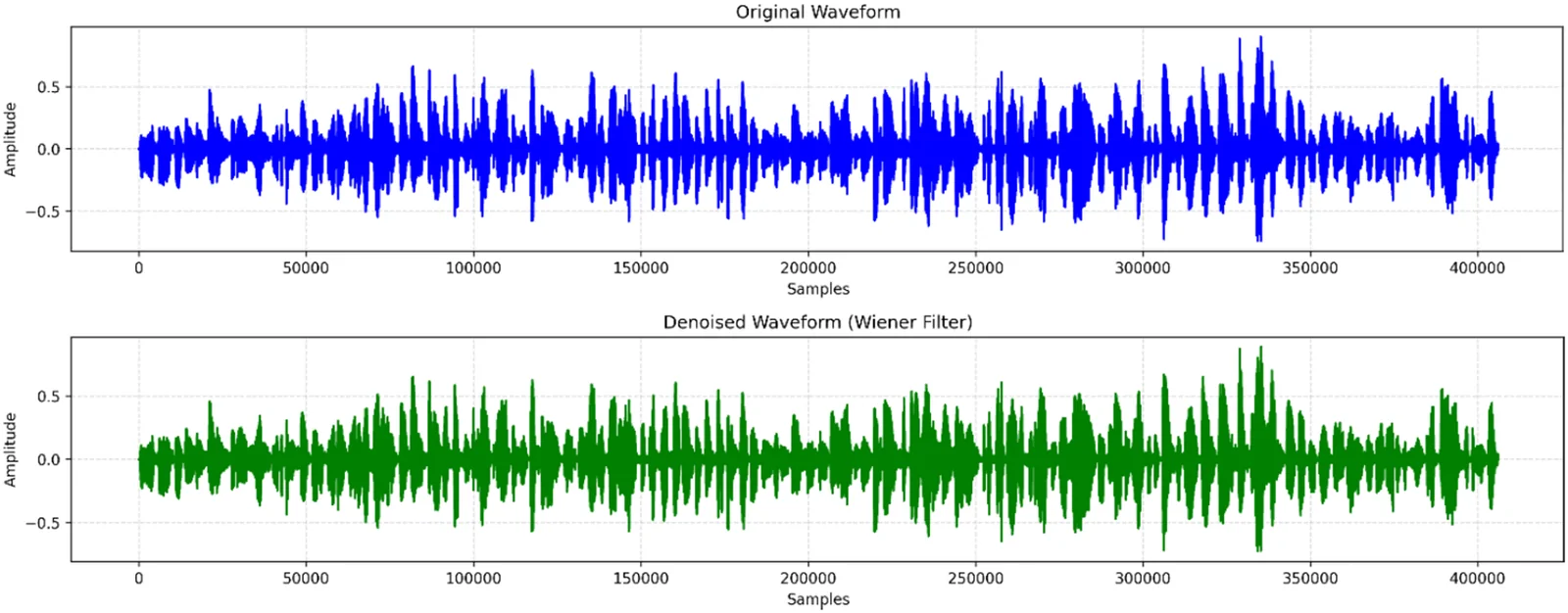
ROC curves of different models.

**Figure 7:**
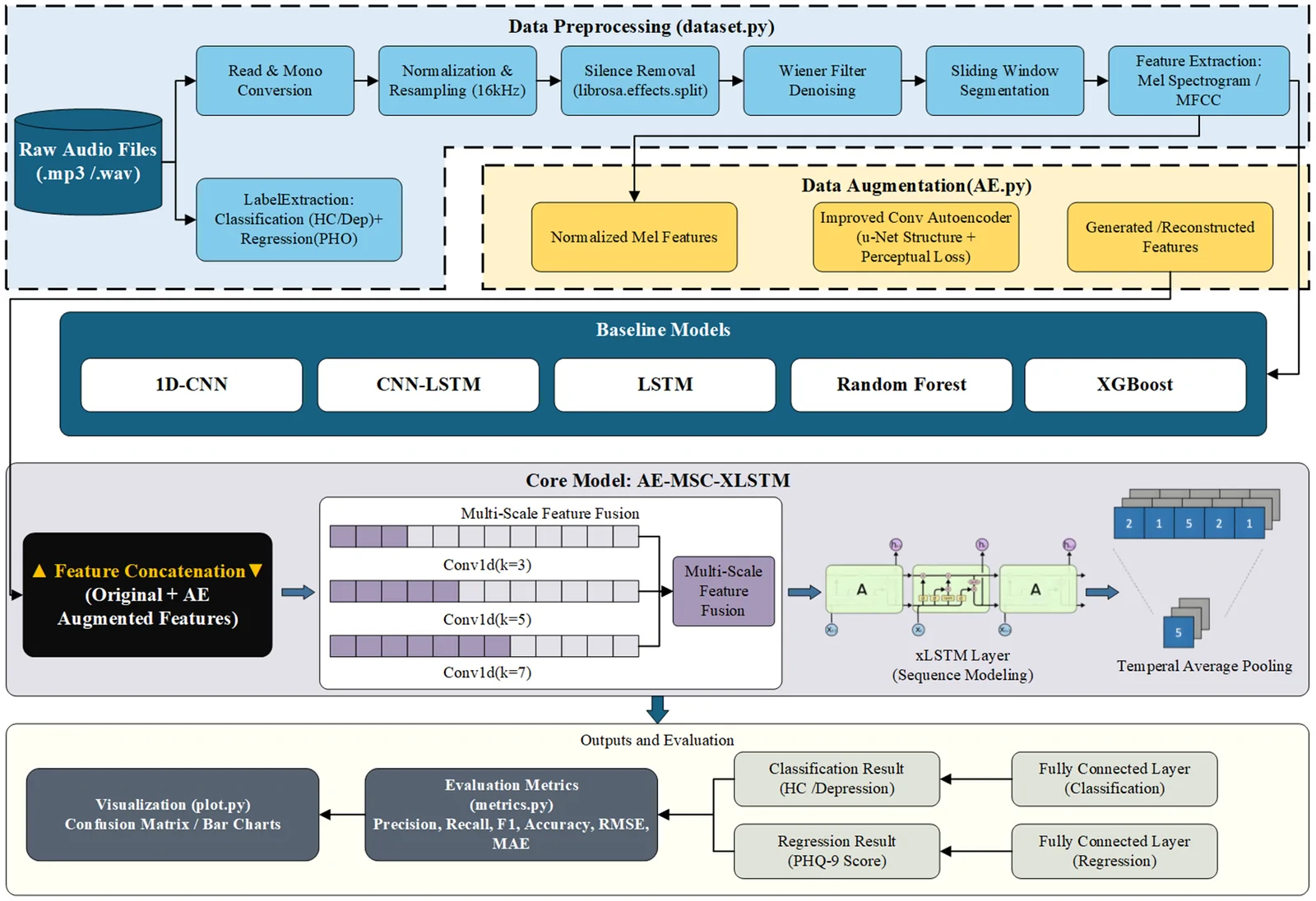
Classification performance of different models.

**Figure 8:**
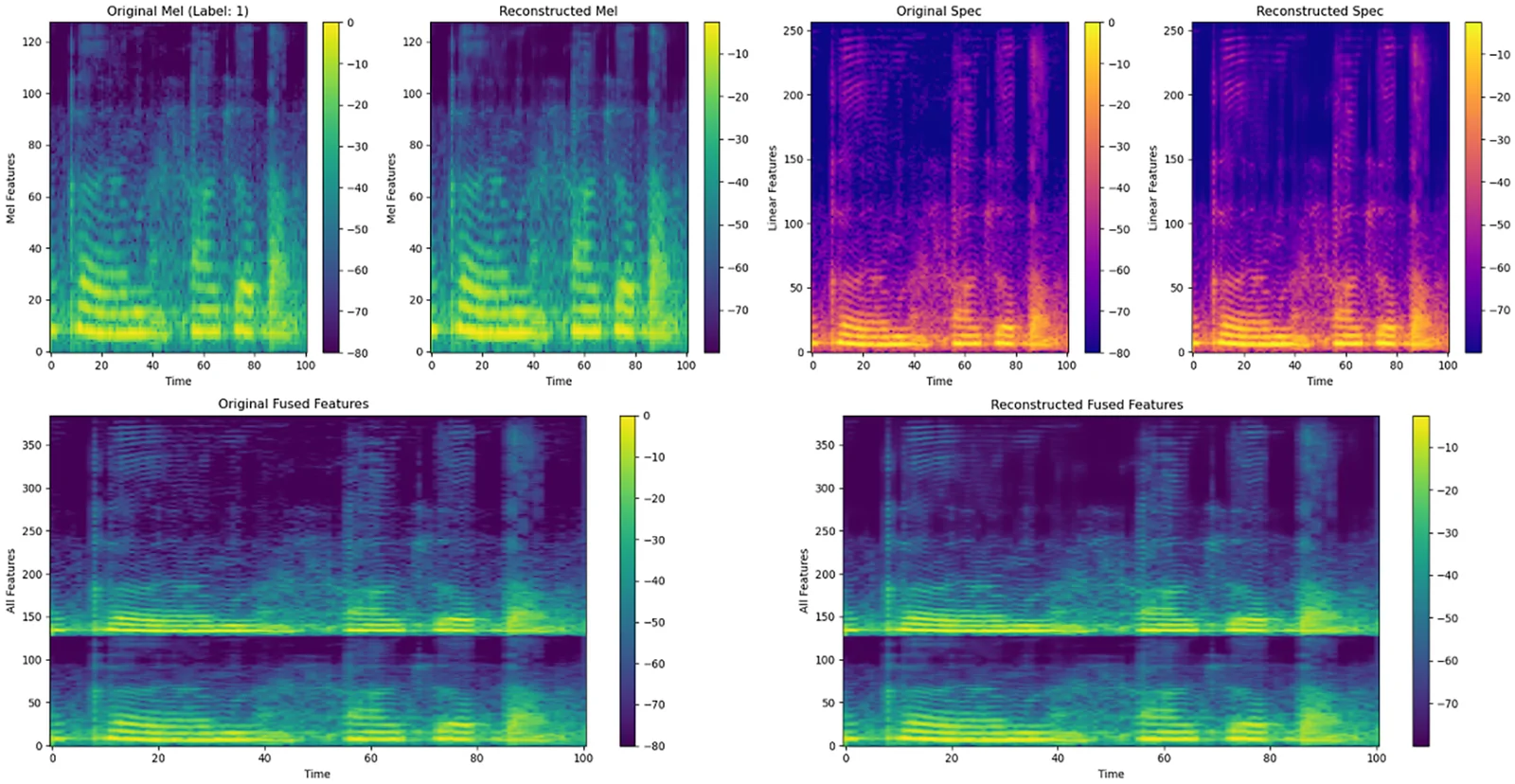
Regression performance metrics of different models.

In the classification task, MAX-Net achieved the highest accuracy (0.901), F1 score (0.902), and AUC (0.945), as shown in **Figures 6** and **7**, indicating that the multi-scale feature modeling and AE architecture introduced in the model can more effectively capture the speech differences across various symptom patterns. For the regression task, MAX-Net achieved the lowest mean absolute error (MAE = 1.610) and the highest coefficient of determination (R² = 0.695), indicating that the model possesses strong severity score prediction capabilities, as shown in **Figure 8**.

### 4.3 Model efficiency

The complexity and inference speeds of each model are shown in **Table 5**. Although MAX-Net employs an integrated architecture, it maintains practical efficiency, with only 0.48M parameters and a single-sample inference time of just 1.82 milliseconds, giving it the potential to support near-real-time applications.

**Table 5:** Efficiency comparison of each model.

Model name	Parameters (M)	Training time (GPU, min)	Inference time (GPU, ms/sample)
Random Forest	0.02	0.46 ± 0.01	0.18 ± 0.27
XGBoost	0.01	0.25 ± 0.03	0.02 ± 0.01
1D CNN	0.05	1.20 ± 0.10	0.06 ± 0.01
CNN–LSTM	0.06	1.60 ± 0.30	0.07 ± 0.01
xLSTM	0.07	38.74 ± 8.98	0.73 ± 0.10
MSC–xLSTM	0.45	38.74 ± 8.98	1.02 ± 0.27
**AE–MSC–** **x** **LSTM**	**0.48**	**42.74 ± 8.98**	**1.82 ± 0.27**

The training and inference times are reported as the mean ± standard deviation over five repeated runs on a 4 × NVIDIA RTX 4090D (24 GB) GPU setup.

Bold values indicate the results of our proposed MAX‑Net model.

### 4.4 Validation on the MODMA dataset

To validate the stability and cross-dataset applicability of the MAX-Net model architecture, we evaluated it on the MODMA dataset using the same model configuration as in HMU-DDAC-24, with retraining on MODMA. With 3,000 AE samples, MAX-Net achieves the best performance among all compared models on the MODMA dataset (AUC = 0.804).

We further compared MAX-Net with representative Transformer-based and self-supervised speech models (AST, Conformer, and Wav2Vec 2.0) on the MODMA dataset. As shown in **Supplementary Table 1**, MAX-Net achieves the best performance (AUC = 0.804), outperforming AST (0.719), Conformer (0.707), and Wav2Vec 2.0 (0.686), while using fewer parameters and requiring no pre-training.

### 4.5 Ablation studies and mechanism analysis

To validate the contribution of each core component in MAX-Net, systematic ablation studies were conducted. Detailed numerical results are summarized in **Supplementary Tables 2**–**6** (see **Supplementary File 1**).

1. xLSTM vs. LSTM

Replacing a vanilla LSTM with xLSTM improved accuracy by 4.6% (0.842 vs. 0.805) and R² by 25.1% (0.494 vs. 0.395),as shown in **Supplementary Table 2**, confirming its superior long-range dependency modeling.

2. Contribution of MSC

The integration of the MSC module brought further gains: compared to the xLSTM baseline (Accuracy=0.842, R²=0.494), it improved classification accuracy to 0.875 and R² to 0.558, while reducing regression MAE to 2.020 (detailed results in **Supplementary Table 3**). This confirms that multi-scale convolutional feature extraction provides richer representations for temporal modeling.

3. Contribution of AE-based augmentation

Integrating the AE module onto the MSC-xLSTM backbone formed the complete MAX-Net, achieving the best performance (Accuracy=0.901, MAE = 1.610, R²=0.695) with minimal added parameters, as detailed in the architecture ablation study (**Supplementary Tables 3**, **5**). Further analysis of data strategies (**Supplementary Table 4**) revealed that training with a mixture of original and AE-generated samples yielded superior results (Accuracy=0.878, R²=0.561) compared to using either type alone. Crucially, this mixed-data strategy substantially reduced the generalization gap from 0.181 to 0.049, demonstrating the AE module’s dual role in feature enhancement and effective augmentation for robust, small-sample learning.

4. Comparative analysis of module contributions

To contextualize the contribution of each component, we compared the performance gains across the three ablation settings. As summarized in **Supplementary Table 6**, xLSTM achieves substantial improvements in both classification accuracy (+4.6%) and R² (+25.1%), demonstrating its particular strength in continuous severity prediction. The MSC module contributes a +3.3% accuracy gain and +11.5% R² improvement, indicating that multi-scale convolutions extract more discriminative local features and facilitate temporal modeling. The AE augmentation, with a minimal parameter overhead of only 0.03M, delivers a notable R² improvement of +24.5% along with a +2.6% accuracy gain. The three modules play distinct yet complementary roles: xLSTM handles long-range temporal modeling, MSC performs multi-scale feature extraction, and AE alleviates data scarcity. Their combination collectively achieves the superior performance of the complete MAX-Net framework.

5. Statistical analysis

To further validate the reliability of our results, we conducted paired t-tests between different data augmentation configurations and the baseline (training with original samples only) on the MODMA dataset. As shown in **Supplementary Table 10**, all AE-based augmentation configurations (AE_3K to AE_28K) achieve statistically significant improvements over the baseline (p< 0.001), whereas conventional augmentation methods (Noise and TimeStretch) show no significant difference (p > 0.05).

It should be noted that these statistical tests are designed to evaluate the effectiveness of the augmentation strategy, rather than to establish the statistical superiority of the complete MAX-Net framework over every competing baseline model. The performance comparisons between MAX-Net and other methods are detailed in **Table 4** (on HMU-DDAC-24) and **Supplementary Table 1** (on MODMA). Together, these complementary evaluations support the conclusion that the performance gains from AE augmentation are not due to random variation and that MAX-Net consistently outperforms the compared methods.

## 5 Discussion

### 5.1 Advancing temporal modeling: the xLSTM advantage

To address the challenge of capturing pathological features in speech associated with depressive symptoms, this paper introduces the extended long short-term memory network (xLSTM) as the core modeling module. Building upon the traditional LSTM, xLSTM incorporates an exponential gating mechanism, which significantly enhances the model’s ability to capture dependencies in long sequences by improving state updates and memory capacity. Chen et al. integrated sLSTM, a variant of xLSTM, into the depressive symptom screening model TTFNet (45). The model outperformed traditional LSTM models significantly on both the DAIC-WOZ and E-DAIC datasets, confirming its potential for screening depressive symptom in long-sequence speech.

This study further validated the superiority of xLSTM through systematic ablation experiments (see S2 in **Supplementary File 1**). With comparable parameter counts and computational costs, xLSTM achieved approximately a 4.7% improvement in classification accuracy (0.842 vs. 0.805) and a 25.1% increase in R² (0.494 vs. 0.395) in regression tasks compared to the baseline LSTM, while maintaining inference times at the millisecond level, thereby achieving a good balance between expressive power and computational efficiency.

### 5.2 The AE module: a unified strategy for robustness and data scarcity

**Figure 9:**
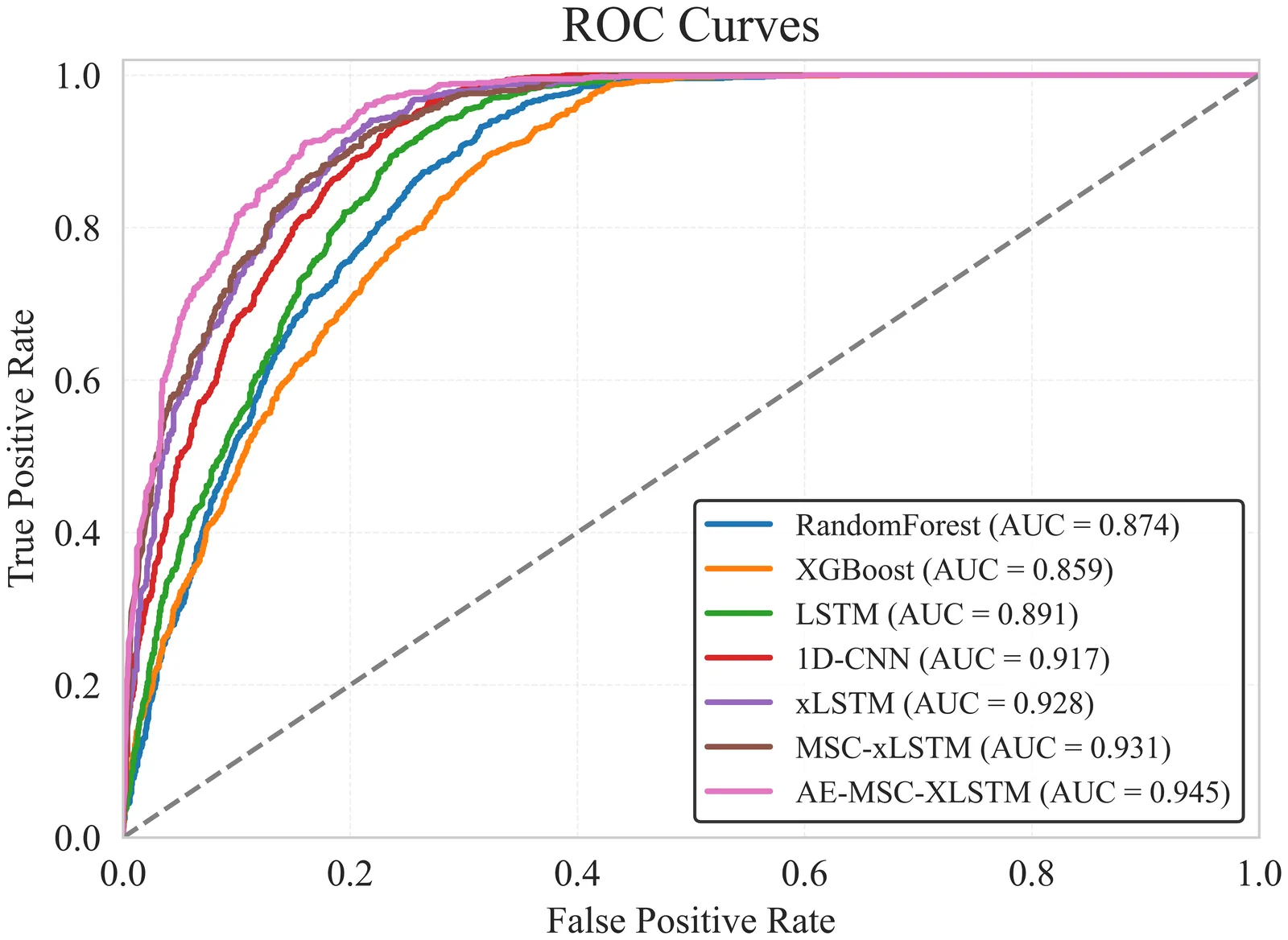
Comparison between original and AE-generated samples.

To address the small sample size issue in depressive symptoms speech data, we designed an improved convolutional AE that employs a U-Net-like architecture for feature enhancement (46, 47). In this experiment, we used the AE to reconstruct depressive symptoms speech features in the latent space. Measurements showed that the original samples and the generated samples exhibited extremely high similarity (cosine similarity = 0.9994; see **Figure 9** for a structural comparison), indicating that the samples generated by the AE retained the detailed features of the original samples.

Previous studies have shown that high-quality data augmentation helps mitigate overfitting under small-sample conditions and improves model generalization (48). In this study, we also observed a similar phenomenon: as shown in **Supplementary Table 4**, under a setting where the model was trained using only AE-generated samples and evaluated on the original test set, it still achieved high performance (Accuracy = 0.869, R² = 0.534). When trained using both original and generated samples, the model’s accuracy improved to 0.878, and the R² value increased to 0.561. Meanwhile, the generalization gap was significantly reduced from 0.181 (when trained solely on original samples) to 0.049. These results demonstrate that the features generated by AE effectively complement the original samples, mitigating overfitting under small-sample conditions and thereby enhancing the model’s robustness.

**Figure 10:**
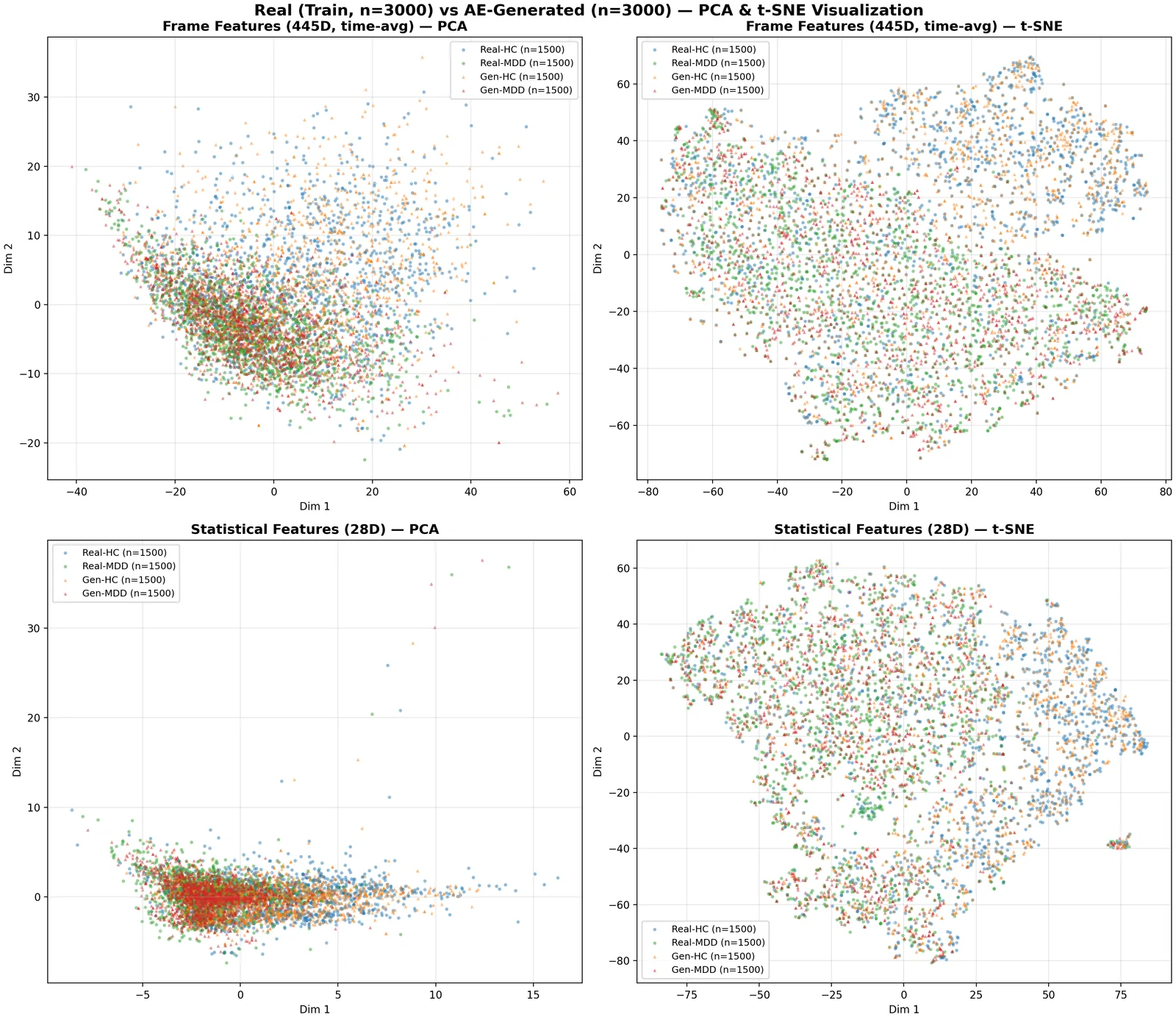
Visualization of original and AE-generated samples in feature space.

To further understand why the AE-generated samples improve generalization, we visualized their feature distribution using PCA and t-SNE (**Figure 10**) and computed quantitative metrics (**Supplementary Table 9**). The results indicate that the generated samples are not merely copies of the originals, but rather form meaningful within-distribution variations that help the model learn a more robust decision boundary.

The sample sizes of the datasets used in this study are relatively small (HMU-DDAC-24 includes 143 participants, and MODMA includes 52). This is not merely a limitation, but rather an objective reality in current psychiatric speech analysis and the core motivation for our proposed architecture. In data-scarce scenarios, a mismatch between model capacity and data size can easily lead to overfitting, resulting in increased variance and compromised robustness on unseen samples. Using generative methods to augment samples can effectively address this issue, as evidenced by the substantial generalization gap (0.181) observed when training solely on original samples (**Supplementary Table 4**). Therefore, our AE-based augmentation is not an optional add-on, but a necessary design tailored for small-sample medical scenarios to enhance model stability.

### 5.3 Effect of the number of generated samples on performance

To investigate the impact of the number of AE-generated samples on model performance and to identify an approximately optimal range for the augmentation ratio, we synthesized up to 30,000 generated samples from the original training data of the two datasets and systematically evaluated the AE-xLSTM model across ten different generation scales ranging from 3,000 to 28,000. The relevant results are summarized in **Supplementary Tables 7** and **S8** in the **Supplementary Materials**.

As shown in **Supplementary Table 7**, as the number of generated samples (NAE) gradually increased from 3,000 to 15,000, the model’s classification and regression performance on the test set both showed an upward trend. When NAE = 15,000 (i.e., the ratio of generated samples to original training samples is approximately 1.85:1), the classification accuracy and F1-score reached 0.882, while the MAE and RMSE in the regression task also dropped to low levels (1.835 and 2.694), at which point the model achieved its best overall performance. When NAE exceeds 15,000, most performance metrics fluctuate or decline slightly, indicating that an excessive number of generated samples can interfere with training. Similarly, validation results on MODMA show that model performance peaks when NAE is 22,000 (with a ratio of generated samples to original training samples of approximately 2:1), followed by a slight decline.

This phenomenon can be explained by the bias-variance trade-off theory (49). Increasing the number of high-quality generated samples expands the training set size to Ntotal = Nreal + NAE, thereby reducing model variance and mitigating overfitting. However, the slight discrepancy between the generated distribution PAE and the true distribution Preal introduces additional distributional bias as the proportion of generated samples α increases. Therefore, theoretically, there exists an optimal ratio α that minimizes the total generalization error. (See **Supplementary File 2** for detailed mathematical derivations.).

In summary, this study experimentally determined the relationship between the number of AE-generated samples and the original training samples and explored its theoretical underpinnings. This finding provides an important reference for the quantitative application of data augmentation strategies in medical speech analysis with small sample sizes.

### 5.4 Limitations

This study aims to provide guidance for the early screening of depressive symptoms using speech-based methods; however, it has the following limitations.

First, the data collected in this experiment is limited in terms of diversity across age, region, and educational background, which may affect the model’s ability to generalize to broader populations. Additionally, the absolute sample sizes of HMU-DDAC-24 (N = 143) and MODMA (N = 52) are modest. While this scale is representative of typical clinical speech studies, and while we have emphasized in Section 5.2 that small-sample conditions constitute the core motivation for our methodological design, larger cohorts would undoubtedly enhance the model’s coverage of population heterogeneity and provide stronger statistical power for subgroup analyses.

Second, the speech data was collected in a quiet laboratory setting, which differs from the variable acoustic environments encountered in daily life; therefore, the model’s effectiveness and robustness in real-world open environments require further investigation.

To address these issues, future work will focus on the following directions:

1. Collaborate across multiple centers and regions to build a speech database with more diverse demographic characteristics to evaluate the model’s effectiveness.
2. Explore effective methods for collecting speech in natural settings to enhance the model’s robustness to complex environmental noise.
3. Integrate multimodal data, such as text and behavioral data, to construct a more comprehensive early-risk assessment model and promote its application in real-world preventive scenarios.
4. Expand the sample size through multi-center collaborations and validate the framework on larger, more diverse cohorts to further substantiate its generalizability and clinical applicability.

## 6 Conclusion

This paper proposes a deep learning framework (MAX-Net) based on speech signals for the automatic screening and assessment of depressive symptoms. Its core innovation lies in the integration of an improved extended long short-term memory network (xLSTM), MSC, and AE feature enhancement, aiming to accurately capture the complex multiscale temporal acoustic features present in depressive speech. The main conclusions are as follows:

1. An efficient and novel architecture for depressive symptom screening is proposed.

To the best of our knowledge, this study is the first to introduce xLSTM into speech-based depressive symptom screening. The resulting AE–MSC–xLSTM model can effectively model both local acoustic abnormalities and long-range contextual dependencies in speech. Experiments on the HMU-DDAC-24 dataset demonstrate that the proposed model achieves excellent performance in the binary classification task, with an F1-score of 0.902 and an AUC of 0.945, and attains a low MAE of 1.610 in depressive severity regression. Its performance is significantly superior to a range of traditional machine learning and deep learning baseline models.

2. Early screening in a high-risk population is explicitly addressed.

Unlike most prior studies that rely on public datasets or clinical patient cohorts, this work conducts an in-depth speech analysis of a non-clinical Chinese university student population. By targeting this high-risk group that is at an early and potentially reversible stage of depressive symptoms, the study provides a low-cost and efficient technical solution for early preliminary screening, thereby creating a valuable time window for subsequent timely interventions.

3. Theoretical and empirical guidance for data augmentation under limited-data conditions is provided.

This study demonstrates the effectiveness of a U-Net–like AE in alleviating data limitations in medical speech analysis. The proposed feature enhancement strategy enlarges the effective training set and improves robustness and generalization, as reflected by a reduction in the generalization gap (from 0.181 to 0.049). Both experimental results and theoretical analyses show that performance is maximized when the ratio of generated to original samples is approximately 1.85:1 on the HMU-DDAC-24 dataset (15,000 generated vs. ~8,000 original) and approximately 2:1 on the MODMA dataset (22,000 generated vs. ~11,000 original), providing a quantitative reference for data augmentation design in limited-data clinical scenarios.

In summary, this work presents a comprehensive framework for speech-based assessment of depressive symptoms. The proposed MAX-Net establishes a promising benchmark for early depressive symptom screening and suggests the potential of AI-driven speech analysis as an accessible mental health tool. Nevertheless, we acknowledge that the current validation, while encouraging, does not yet support clinical deployment without further large-scale external validation. Future work will focus on multimodal information fusion and on validating the framework’s real-world applicability through large-scale, multi-center clinical studies.

## Data Availability

The raw data supporting the conclusions of this article will be made available by the authors, without undue reservation.
