# Supplementary material for: Speech-based early depressive symptom screening using MAX-Net: a multi-scale autoencoder-augmented xLSTM framework
Source: Front Psychiatry. 2026 Sep 3;17:1907554. doi: 10.3389/fpsyt.2026.1907554 (PMC13584913; doi:10.3389/fpsyt.2026.1907554)
Supplement: Supplementary file 1 [file Table1.docx]

| **Model name** |  | **Accuracy** | **Precision** | **Recall** | **F1-Socre** | **AUC** | **MAE** | **RMSE** | **Params** |  |  |
| --- | --- | --- | --- | --- | --- | --- | --- | --- | --- | --- | --- |
| Random Forest |  | 0.494 ± 0.006 | 0.580 ± 0.005 | 0.494 ± 0.006 | 0.510 ± 0.007 | 0.530 ± 0.009 | 8.160 ± 0.017 | 9.146 ± 0.022 | 0.02M |  |  |
| XGBoost |  | 0.518 ± 0.008 | 0.588 ± 0.006 | 0.518 ± 0.008 | 0.534 ± 0.008 | 0.539 ± 0.007 | 7.840 ± 0.041 | 8.789 ± 0.046 | 0.01M |  |  |
| LSTM |  | 0.525 ± 0.028 | 0.603 ± 0.009 | 0.525 ± 0.028 | 0.539 ± 0.030 | 0.583 ± 0.022 | 7.917 ± 0.526 | 9.869 ± 0.513 | 0.008M |  |  |
| Wav2Vec 2.0 |  | 0.619 ± 0.015 | 0.627 ± 0.014 | 0.619 ± 0.015 | 0.611 ± 0.020 | 0.686 ± 0.021 | 5.744 ± 0.195 | 8.275 ± 0.398 | 1.61M |  |  |
| xLSTM |  | 0.628 ± 0.019 | 0.632 ± 0.017 | 0.628 ± 0.019 | 0.622 ± 0.023 | 0.690 ± 0.039 | 5.280 ± 0.197 | 7.352 ± 0.390 | 0.07M |  |  |
| Conformer |  | 0.644 ± 0.027 | 0.656 ± 0.034 | 0.644 ± 0.027 | 0.637 ± 0.027 | 0.707 ± 0.037 | 6.021 ± 0.429 | 8.310 ± 0.475 | 1.66M |  |  |
| AST |  | 0.655 ± 0.021 | 0.667 ± 0.017 | 0.655 ± 0.021 | 0.646 ± 0.025 | 0.719 ± 0.034 | 5.697 ± 0.350 | 8.059 ± 0.407 | 0.65M |  |  |
| Zero-shot(HMU**→**MODMA) |  | 0.554 | 0.573 | 0.554 | 0.512 | 0.586 | 7.996 | 10.688 | 0.48M |  |  |
| AE + xLSTM |  | 0.762 ± 0.013 | 0.780 ± 0.022 | 0.762 ± 0.013 | 0.757 ± 0.011 | 0.791 ± 0.006 | 3.625 ± 0.187 | 4.773 ± 0.256 | 0.45M |  |  |
| **MAX-Net** |  | **0.769 ± 0.011** | **0.784 ± 0.02** | **0.769 ± 0.011** | **0.765 ± 0.011** | **0.804 ± 0.014** | **3.410 ± 0.211** | **4.522 ± 0.273** | **0.48M** |  |  |

**Table S1: Cross-dataset performance comparison on MODMA**

Note:AE + xLSTM and MAX-Net were trained with 3,000 AE samples.

**Table S2. LSTM Architecture Selection**

| **Model name** | **Parameters (M)** | **Training Time (GPU, min)** | **Inference Time (GPU, ms/sample）** | **Accuracy** | **F1-score** | **R²** |  |
| --- | --- | --- | --- | --- | --- | --- | --- |
| LSTM | 0.008 | 4.93 ± 1.12 | 0.352 ± 0.543 | 0.805 ± 0.003 | 0.812 ± 0.003 | 0.395 ± 0.007 |  |
| sLSTM | 0.06 | 38.09 ± 10.07 | 0.489 ± 0.269 | 0.810 ± 0.007 | 0.806 ± 0.007 | 0.367 ± 0.024 |  |
| xLSTM | 0.07 | 38.747 ± 8.98 | 0.731 ± 0.103 | 0.842 ± 0.005 | 0.842 ± 0.005 | 0.494 ± 0.017 |  |

Note: All training and inference times were measured on a GPU environment (4 × NVIDIA RTX 4090D, 24 GB). Reported values are mean ± standard deviation over five repeated experiments.

**Table S3. Progressive Model Architecture Ablation Study for Classification and Regression Tasks**

| **Model Architecture** | **Classification Performance** | | | | **Regression Performance** | | | |  |  |
| --- | --- | --- | --- | --- | --- | --- | --- | --- | --- | --- |
|  | **Accuracy** | **Precision** | **Recall** | **F1-score** | **MAE** | **RMSE** | **R² Score** |  |  |  |
| LSTM | 0.805 ± 0.003 | 0.819 ± 0.006 | 0.805 ± 0.003 | 0.812 ± 0.003 | 2.579 ± 0.022 | 3.422 ± 0.036 | 0.395 ± 0.007 |  |  |  |
| xLSTM (2 layers) | 0.842 ± 0.005 | 0.843 ± 0.006 | 0.842 ± 0.005 | 0.842 ± 0.005 | 2.288 ± 0.060 | 3.154 ± 0.052 | 0.494 ± 0.017 |  |  |  |
| +MSC (MSC + xLSTM) | 0.875 ± 0.005 | 0.882 ± 0.006 | 0.874 ± 0.005 | 0.878 ± 0.005 | 2.020 ± 0.085 | 2.672 ± 0.127 | 0.558 ± 0.029 |  |  |  |
| +AE Blocks (MAX-Net) | **0.901 ± 0.004** | **0.906 ± 0.005** | **0.907 ± 0.004** | **0.902 ± 0.004** | **1.610 ± 0.022** | **2.310 ± 0.100** | **0.695 ± 0.027** |  |  |  |

**Table S4. Ablation Study on Data Augmentation Strategies**

| **Data Strategy** | **Training Samples** | **Accuracy** | **F1-score** | **R²** | **Generalization Gap** | **Training Time (GPU, min)** | **Inference Times (GPU, ms/sample）** |
| --- | --- | --- | --- | --- | --- | --- | --- |
| Original Samples | 8,132 | 0.842 ± 0.005 | 0.842 ± 0.005 | 0.494 ±0.017 | 0.181 ± 0.005 | 38.737 ± 8.986 | 0.731 ± 0.103 |
| Generated Samples | 6,000 | 0.869 ± 0.006 | 0.870 ± 0.006 | 0.534 ±0.008 | 0.122 ± 0.006 | 15.943 ± 2.840 | 0.277 ± 0.009 |
| Original + Generated Samples | 14,132 | 0.878 ± 0.007 | 0.879 ± 0.007 | 0.561 ±0.010 | 0.049 ± 0.003 | 135.736 ± 17.911 | 1.629 ± 0.214 |

**Note**: From comparability of training time, 6,000 AE generated samples were used for this experiment. Reported values are the mean ± standard deviation over five runs. The model used is the AE–xLSTM.

**Table S5. Methods Comparison on Our Dataset**

| **Model name** | **Accuracy** | **F1-score** | **R²** | **MAE** | **AUC** | **Parameters**  **(M)** | **Training Time**  **(GPU, min)** | **Inference Time**  **(GPU, ms/sample)** |
| --- | --- | --- | --- | --- | --- | --- | --- | --- |
| Random Forest | 0.781 ± 0.009 | 0.780 ± 0.009 | 0.398 ± 0.008 | 2.594 ± 0.024 | 0.874 ± 0.006 | 0.02 | 0.45 ± 0.05 | 0.176 ± 0.027 |
| XGBoost | 0.741 ± 0.008 | 0.741 ± 0.009 | 0.267 ± 0.004 | 2.926 ± 0.037 | 0.859 ± 0.007 | 0.01 | 0.25 ± 0.02 | 0.012 ± 0.001 |
| 1D-CNN | 0.844 ± 0.005 | 0.846 ± 0.005 | 0.427 ± 0.016 | 2.580 ± 0.036 | 0.917 ± 0.001 | 0.05 | 1.20 ± 0.20 | 0.006 ± 0.010 |
| CNN–LSTM | 0.856 ± 0.003 | 0.886 ± 0.003 | 0.465 ± 0.012 | 2.367 ± 0.044 | 0.920 ± 0.003 | 0.06 | 1.60 ± 0.30 | 0.070 ± 0.001 |
| MSC–xLSTM | 0.875 ± 0.005 | 0.878 ± 0.005 | 0.558 ± 0.029 | 2.020 ± 0.085 | 0.931 ± 0.005 | 0.45 | 38.74 ± 8.98 | 0.731 ± 0.103 |
| **AE-MSC–xLSTM (MAX-Net)** | **0.901 ± 0.004** | **0.902 ± 0.004** | **0.695 ± 0.027** | **1.610 ± 0.022** | **0.945 ± 0.003** | **0.48** | **42.74 ± 8.98** | **0.750 ± 0.110** |

**Note:** All training and inference times were obtained in a GPU environment (4 × NVIDIA RTX 4090D, 24 GB). Each result represents the mean ± standard deviation over five repeated experiments.

**Table S6. Summary of module-wise contributions to classification and regression performance**

| Module Addition | Accuracy Δ | R² Δ | Parameter Increase |
| --- | --- | --- | --- |
| LSTM → xLSTM | +4.6% (0.805→0.842) | +25.1% (0.395→0.494) | +0.06M |
| xLSTM → +MSC | +3.9% (0.842→0.875) | +13.0% (0.494→0.558) | +0.38M |
| MSC-xLSTM → +AE | +3.0% (0.875→0.901) | +24.6% (0.558→0.695) | +0.03M |

**Table S7. Effect of Different Numbers of AE-Generated Samples on Model Performance**

| **N_AE_** | **Accuracy** | **F1-score** | **Precision** | **Recall** | **MAE** | **RMSE** |
| --- | --- | --- | --- | --- | --- | --- |
| 0 | 0.842 ± 0.005 | 0.842 ± 0.005 | 0.843 ± 0.006 | 0.842 ± 0.005 | 2.288 ± 0.060 | 3.154 ± 0.052 |
| Noise | 0.830 ± 0.024 | 0.832 ± 0.026 | 0.840 ± 0.028 | 0.840 ± 0.024 | 2.521 ± 0.136 | 7.306 ± 0.259 |
| TimeStretch | 0.836 ± 0.022 | 0.831 ± 0.023 | 0.841 ± 0.024 | 0.836 ± 0.022 | 2.635 ± 0.401 | 7.520 ± 0.540 |
| AE_3K | 0.871 ± 0.004 | 0.871 ± 0.004 | 0.871 ± 0.004 | 0.867 ± 0.025 | 2.883 ± 0.035 | 0.871 ± 0.004 |
| AE_6K | 0.872 ± 0.004 | 0.872 ± 0.004 | 0.873 ± 0.004 | 0.872 ± 0.004 | 1.916 ± 0.025 | 2.881 ± 0.035 |
| AE_12K | 0.878 ± 0.004 | 0.878 ± 0.004 | 0.879 ± 0.004 | 0.878 ± 0.004 | 1.840 ± 0.025 | 2.691 ± 0.035 |
| **AE_15K** | **0.882 ± 0.004** | **0.882 ± 0.004** | **0.882 ± 0.004** | **0.882 ± 0.004** | **1.835 ± 0.025** | **2.694 ± 0.035** |
| AE_18K | 0.876 ± 0.004 | 0.876 ± 0.004 | 0.876 ± 0.004 | 0.876 ± 0.004 | 1.875 ± 0.025 | 2.713 ± 0.035 |
| AE_22K | 0.877 ± 0.004 | 0.877 ± 0.004 | 0.877 ± 0.004 | 0.877 ± 0.004 | 1.867 ± 0.025 | 2.760 ± 0.03 |
| AE_28K | 0.875 ± 0.004 | 0.875 ± 0.004 | 0.876 ± 0.004 | 0.875 ± 0.004 | 1.851 ± 0.025 | 2.708 ± 0.035 |

Note: N_AE_ denotes the number of AE-generated training samples; all values are reported on the test set.

**Table S8. Results of different AE-generated sample size on the MODMA dataset**

| N_AE_ | Accuracy | Precision | Recall | F1-Score | AUC | MAE | RMSE |
| --- | --- | --- | --- | --- | --- | --- | --- |
| 0 | 0.637 ± 0.025 | 0.640 ± 0.026 | 0.637 ± 0.025 | 0.634 ± 0.026 | 0.710 ± 0.036 | 5.568 ± 0.368 | 7.345 ± 0.535 |
| Noise | 0.640 ± 0.024 | 0.650 ± 0.028 | 0.640 ± 0.024 | 0.632 ± 0.026 | 0.709 ± 0.036 | 5.521 ± 0.136 | 7.306 ± 0.259 |
| TimeStretch | 0.636 ± 0.022 | 0.643 ± 0.024 | 0.636 ± 0.022 | 0.631 ± 0.023 | 0.715 ± 0.029 | 5.635 ± 0.401 | 7.520 ± 0.540 |
| AE_3K | 0.761 ± 0.013 | 0.780 ± 0.022 | 0.761 ± 0.013 | 0.757 ± 0.011 | 0.790 ± 0.006 | 3.625 ± 0.187 | 4.773 ± 0.256 |
| AE_6K | 0.792 ± 0.003 | 0.818 ± 0.008 | 0.792 ± 0.003 | 0.787 ± 0.003 | 0.804 ± 0.012 | 3.034 ± 0.165 | 3.995 ± 0.171 |
| AE_12K | 0.806 ± 0.009 | 0.837 ± 0.015 | 0.806 ± 0.009 | 0.801 ± 0.009 | 0.801 ± 0.015 | 2.660 ± 0.102 | 3.486 ± 0.157 |
| AE_15K | 0.806 ± 0.010 | 0.835 ± 0.017 | 0.806 ± 0.010 | 0.801 ± 0.010 | 0.803 ± 0.023 | 2.745 ± 0.299 | 3.619 ± 0.441 |
| AE_18K | 0.817 ± 0.005 | 0.853 ± 0.008 | 0.817 ± 0.005 | 0.811 ± 0.005 | 0.801 ± 0.008 | 2.572 ± 0.251 | 3.302 ± 0.322 |
| **AE_22K** | **0.818 ± 0.003** | **0.855 ± 0.006** | **0.818 ± 0.003** | **0.812 ± 0.003** | **0.807 ± 0.012** | **2.484 ± 0.142** | **3.204 ± 0.166** |
| AE_28K | 0.817 ± 0.007 | 0.854 ± 0.013 | 0.817 ± 0.007 | 0.812 ± 0.007 | 0.804 ± 0.014 | 2.420 ± 0.173 | 3.149 ± 0.267 |

**Table S9. Distribution comparison between original and AE-generated samples.**

| **Metric** | **Frame-level features (445D)** | **Statistical features (28D)** | **Interpretation** |
| --- | --- | --- | --- |
| C2ST accuracy | 0.6875 | 0.615 | Values closer to 0.5 indicate that real and generated samples are harder to distinguish, implying highly similar distributions. |
| Precision | 0.949 | 0.999 | Proportion of generated samples falling within the original distribution support |
| Coverage | 0.967 | 0.971 | Proportion of original samples covered by generated samples |
| Density | 1.265 | 2.042 | ≈1 indicates generated samples are uniformly distributed around original samples |
| MMD² | 0.0148 | 0.0031 | Smaller value indicates closer distributions |

**Table S10. Statistical significance tests between different augmentation configurations and baseline on MODMA**

| **Config** | **Metric** | **Baseline Mean** | **Config Mean** | **t-statistic** | **p-value (t-test)** | **Significant** |
| --- | --- | --- | --- | --- | --- | --- |
| Noise | Accuracy | 0.637 | 0.640 | 0.25 | 0.812 | No |
| TimeStretch | Accuracy | 0.637 | 0.636 | -0.06 | 0.957 | No |
| AE_3K | Accuracy | 0.637 | 0.761 | 8.71 | **<0.001** | **Yes** |
| AE_6K | Accuracy | 0.637 | 0.792 | 12.22 | **<0.001** | **Yes** |
| AE_12K | Accuracy | 0.637 | 0.806 | 10.46 | **<0.001** | **Yes** |
| AE_15K | Accuracy | 0.637 | 0.806 | 9.75 | **<0.001** | **Yes** |
| AE_18K | Accuracy | 0.637 | 0.817 | 13.17 | **<0.001** | **Yes** |
| AE_22K | Accuracy | 0.637 | 0.818 | 16.58 | **<0.001** | **Yes** |
| AE_28K | Accuracy | 0.637 | 0.817 | 11.33 | <0.001 | Yes |

**Note:** All values are derived from test set results over five repeated runs. p-values are based on paired t-tests (df=4). Baseline Mean and Config Mean are consistent with Table S8. Bold values indicate statistical significance (p < 0.05).
