# Supplementary material for: Speech-based early depressive symptom screening using MAX-Net: a multi-scale autoencoder-augmented xLSTM framework
Source: Front Psychiatry. 2026 Sep 3;17:1907554. doi: 10.3389/fpsyt.2026.1907554 (PMC13584913; doi:10.3389/fpsyt.2026.1907554)
Supplement: Supplementary file 2 [file Table2.docx]

**Supplementary File 2. Theoretical derivation and analysis of the optimal number of AE-generated samples**

This supplementary file presents a theoretical analysis of AE-based data augmentation using the bias–variance decomposition, including problem formulation, modeling of bias and variance terms under mixed real and generated training data, and derivation of the optimal generation ratio that minimizes expected generalization error.

**1 Optimal generation ratio model based on the bias–variance trade-off**

**1.1 Problem formulation**

We make the following assumptions:

1. Real training set: D_real_ = {(x_i,y_i)},i=1,...,N

2. Generated training set：D_AE_ = $\{(\tilde{x}\_j,\tilde{y\_}j)\},$j=1,…,M

3. Generation ratio：α= M/N

4. Total size of the mixed training set：N_total_ = N + M = N(1+α)

Considering a supervised learning task and assuming a squared loss function, the expected generalization error of the model can be decomposed as

$E\left[ (y-\grave{f}(x))^{2} \right]=\mathrm{Bias}^{2}(\grave{f})+Var(\grave{f})+\sigma^{2}$，

where $\grave{f}$ denotes the model trained on *D_mix_*，and $\sigma^{2}$ represents the inherent noise in the data.

**1.2 Modeling the bias term**

Let *P_real_* denote the real data distribution and P_AE_ the distribution of AE-generated data. We introduce a distributional distance: D=d(P_AE_,P_real_). As the generation ratio α increases, the effective training distribution deviates further from *P_real_*, thereby increasing the bias. For analytical simplicity, within a small-to-moderate range of α, we approximate

$$\mathrm{Bias}^{2}(\alpha)\approx\lambda_{1}\cdot\alpha\cdot D$$

where λ_1_ is a task-dependent constant.

**1.3 Modeling the variance term**

The variance term reflects the model’s sensitivity to randomness in the training samples. Following classical learning theory, we assume

$$\mathrm{Var}(\alpha)\approx\lambda_{2}\cdot\frac{C}{N_{\text{total }}}=\lambda_{2}\cdot\frac{C}{N(1+\alpha)},$$

where *C* denotes model complexity (e.g., the effective number of parameters), and *λ_2_* is a constant.

**1.4 Total error function and optimal generation ratio**

By combining the bias and variance terms and ignoring the constant noise term, the total generalization error can be approximated as

$$E(\alpha)\approx\lambda_{1}\alpha D+\frac{\lambda_{2}C}{N(1+\alpha)}.$$

Taking the derivative of *E(α)* with respect to α and setting it to zero yields

$$\frac{dE}{d\alpha}=\lambda_{1}D-\frac{\lambda_{2}C}{N(1+\alpha)^{2}}=0.$$

Let k=λ_1_/λ_2_. The optimal generation ratio α* is then given by

$$\alpha^{*}=\sqrt{\frac{\lambda_{2}C}{\lambda_{1}DN}}-1=\sqrt{\frac{C}{kDN}}-1.$$

Furthermore, since α ≥ 0, N > 0, C > 0, and λ_2_ > 0, we have (1+α)^3^ > 0. The second derivative is

$$\frac{d^{2}E}{d\alpha^{2}}=\frac{2\lambda_{2}C}{N(1+\alpha)^{3}}>0,$$

indicating that the solution corresponds to a local minimum.

**2 Factor analysis**

**2.1 Analysis based on partial derivatives**

Since

$$\alpha^{*}=\sqrt{\frac{C}{kDN}}-1,$$

the optimal generation ratio is jointly determined by three factors: *D*, *C*, and *N*.

*1. Effect of generation quality (D)*

Taking the partial derivative of α^∗^ with respect to D yields

$$\frac{\partial\alpha^{*}}{\partial D}=\frac{\partial}{\partial D}\left( \sqrt{\frac{C}{kDN}}-1 \right)=-\frac{1}{2}\sqrt{\frac{C}{kN}}D^{-\frac{3}{2}}<0.$$

This indicates that a larger discrepancy between the generated and real data distributions (i.e., larger D, corresponding to lower generation quality) leads to a smaller optimal generation ratio α^∗^.

*2. Effect of model complexity (C)*

Taking the partial derivative of α^∗^ with respect to *C* yields

$$\frac{\partial\alpha^{*}}{\partial C}=\frac{\partial}{\partial C}\left( \sqrt{\frac{C}{kDN}}-1 \right)=\frac{1}{2}\frac{1}{\sqrt{kDN}}C^{-\frac{1}{2}}>0.$$

This indicates that as model complexity increases, the demand for training data grows, resulting in a larger optimal generation ratio α^∗^.

*3. Effect of the number of real samples (N)*

Taking the partial derivative of α^∗^with respect to N yields

$$\frac{\partial\alpha^{*}}{\partial N}=\frac{\partial}{\partial N}\left( \sqrt{\frac{C}{kDN}}-1 \right)=-\frac{1}{2}\sqrt{\frac{C}{kD}}N^{-\frac{3}{2}}<0.$$

This indicates that as the number of real samples *N* increases, the marginal benefit of additional generated data decreases, and the optimal generation ratio α^∗^ correspondingly decreases.

**2.2 Relation to the experiments in this study**

In this paper, the experimental settings are as follows:

1. The number of training samples in HMU-DDAC-24 is N_1_ ≈ 8000, and the experimentally observed optimal generation ratio is α* ≈ 1.85 (i.e., the number of generated samples M_1_ ≈ 15,000)

2. The number of trainning samples in MODMA is N_2_ ≈ 11,000, and the experimentally observed optimal generation ratio is α* ≈ 2.0 (i.e., the number of generated samples M_2_ ≈ 22,000)

3. The KL divergence between the generated and real distributions is approximately 0.024, indicating relatively high generation quality.

4. The proposed MAX-Net model exhibits relatively high model complexity.

Substituting N = 8000, D ≈ 0.024, and $\alpha^{\star}$ ≈ 1.5 into the derived relationship, we obtain the implicit ratio between effective model complexity and the task-related constant:

$$\frac{C}{k}=\left( \alpha^{*}+1 \right)^{2}\times D\times N\approx1600.$$

And for MODMA :

$$\frac{C}{k}=\left( \alpha^{*}+1 \right)^{2}\times D\times N\approx2300.$$

This result indicates that, under the current task and model configuration, the effective strength of “increasing samples to reduce variance” on the model side (reflected by C/k) is significantly greater than the bias pressure introduced by the distributional shift D. Given that D itself is relatively small (indicating high generation quality), this can be interpreted as follows: in our experimental setting, moderately increasing the proportion of high‑quality generated samples indeed primarily helps to reduce variance, while the bias introduced thereby remains within an acceptable range.

It should be noted that the above derivation is primarily intended to describe the qualitative trends and relative relationships that influence the optimal generation ratio, rather than to provide a "closed‑form formula" that is universally applicable across all tasks. In practice, the constants C, D, and k are difficult to estimate accurately; therefore, the optimal ratio should still be determined empirically by validating performance over a set of candidate α values.

**3 References for selecting the optimal generation ratio**

**3.1 Theoretical selection procedure**

Building on the bias–variance analysis, we propose a theory-guided procedure for selecting the generation ratio:

*1. Assess generation quality*

Use a distributional distance to measure the discrepancy between the generated P_AE_ and the real P_real_ distributions :

$$D=\mathrm{KL}\left( P_{\mathrm{AE}}\|P_{\text{real }} \right)$$

*2. Assess model complexity*

Adopt empirical indicators that are positively correlated with the theoretical complexity term *C,* such that:

$$C\propto\text{ (number of parameters) }\times\text{ (model depth) × (degree of nonlinearity ) .}$$

*3. Compute an initial theoretical ratio*

Based on the theoretically derived expression for the optimal generation ratio, obtain an initial estimate:

$$\alpha_{0}=\sqrt{\frac{C}{kDN}}-1,k=\frac{\lambda_{1}}{\lambda_{2}},$$

where *N* is the number of real samples, *D* denotes the generation quality indicator, and *k* reflects the relative weight between the bias and variance terms.

*4. Local refinement and validation*

Centered at α_0_, perform a small-range grid search in its neighborhood (e.g.,α∈[$\alpha_{0}$−0.5,$\alpha_{0}$+0.5]), evaluate performance on a validation set, and select the empirically optimal generation ratio α^∗^.

**3.2 Empirical feference interval**

In practical applications, it is often difficult to accurately compute quantities such as *C* and *k*. Therefore, based on the above theoretical trends derived above, we provide a more operational, empirical recommendation range for the generation ratio α (see the table below).

| **Scenario characteristics** | **Recommended generation ratio range α** | **Theoretical rationale (bias–variance perspective)** |
| --- | --- | --- |
| High-quality generation（D < 0.01）+ complex model（$C>{10}^{6}$） | α≈1.0–2.0 | The generated data closely match the real distribution, introducing minimal bias. Complex models typically exhibit high variance and strong sensitivity to sample size; increasing the generation ratio effectively reduces variance. |
| Medium-quality generation（0.01≤ D <0.05）+ medium-complexity model | α ≈0.5–1.0 | The generated data show moderate distributional shift, requiring a balance between bias control and variance reduction. A moderate generation ratio often yields improved overall performance. |
| Low-quality generation（D ≥ 0.05）+ simple model（$C<{10}^{5}$） | α< 0.5 | Simple models inherently have low variance, while the generated data introduce substantial distribution bias. Increasing the generation ratio may cause bias to dominate, so the proportion of generated samples should be limited. |

**Note:** The above ranges are not strictly derived from theory, but are empirical recommendations obtained by combining theoretical trends with experimental observations. For specific tasks, it is advisable to further refine them using the *α_0_* calculation and local search procedure described in Section 3.1.
